# Nab-paclitaxel versus paclitaxel for taxane acute pain syndrome in solid tumors: a systematic review and meta-analysis

**DOI:** 10.3389/fonc.2025.1650191

**Published:** 2026-01-14

**Authors:** Lai Wei, HongBo Li, ZhiYong Wang

**Affiliations:** 1 Department of Surgery, Affillated Hospital of Beihua University, Jilin, Jilin, China; 2 Department of Breast and Thyroid Surgery, Jinhua People’s Hospital, Jinhua, ZheJiang, China

**Keywords:** meta-analysis, myalgia, nab-paclitaxel, paclitaxel, taxane acute pain syndrome

## Abstract

**Objective:**

To quantitatively compare the incidence and symptom-specific risk of taxane-associated acute pain syndrome (TAPS) between nab-paclitaxel (nab-PTX) and paclitaxel (PTX) in adults with solid tumors, with pre-specified stratification by dosing frequency.

**Methods:**

We systematically searched PubMed, Embase and Cochrane Library (to November 30, 2023) for randomised trials comparing nab-PTX versus PTX. After excluding docetaxel and other taxanes, nine head-to-head RCTs (3,699 patients) were pooled using random-effects models. Odds ratios (OR) and risk ratios (RR) were calculated for arthralgia and myalgia, with subgroup analyses by q3w, q4w and weekly schedules. Risk of bias was assessed with RoB 2.0 and publication bias by funnel plots and Egger’s test.

**Results:**

Myalgia incidence was significantly higher with nab-PTX (OR 1.25, 95% CI 1.06--1.48; I² = 0%), whereas arthralgia did not differ (OR 1.07, 0.91–1.25; I² = 0%). The excess myalgia was confined to the every-4-week (q4w, days 1,8,15) schedule (OR 1.32, 1.09–1.59; interaction p = 0.010), with no signal in q3w or weekly regimens. Up to one-quarter of q4w nab-PTX recipients experienced moderate-to-severe myalgia. Results were robust across sensitivity analyses and showed no publication bias.

**Conclusions:**

Nab-PTX selectively increases acute myalgia risk by 25% versus PTX, exclusively within the q4w schedule, without affecting arthralgia. Dosing frequency, not cumulative dose, drives this symptom-specific toxicity. Clinicians should consider schedule selection and proactive analgesia when prescribing nab-PTX. https://www.crd.york.ac.uk/PROSPERO

**Systematic Review Registration:**

https://www.crd.york.ac.uk/PROSPERO, identifier CRD42023484814.

## 1 Introduction

Paclitaxel is a microtubule-stabilizing taxane widely used in the treatment of breast, lung, gastric, and urothelial cancers (1–5). Currently, several paclitaxel formulations are available in clinical practice, including the conventional Cremophor-based formulation, albumin-bound paclitaxel, and polymeric micelle–based formulations. Cremophor-free formulations such as nab-paclitaxel eliminate the need for Cremophor and provide greater exposure to unbound, pharmacologically active paclitaxel (6). The two formulations have comparable efficacy but different toxicity profiles. One clinically important yet often overlooked toxicity is the taxane-associated acute pain syndrome (TAPS), which manifests as arthralgia or myalgia occurring within 24–48 hours after infusion and lasting for about 5–7 days. It can reduce quality of life, affect treatment adherence, and even lead to dose reductions or early discontinuation of treatment (7–9).

Although TAPS has been recognized clinically, there is still a lack of quantitative evidence regarding the relative incidence of TAPS between nab-PTX and PTX. The only previous meta-analysis combined docetaxel with paclitaxel formulations and concluded that paclitaxel had a higher pain risk, but this confounded the drug-specific toxicity mechanisms (10). Since the publication of that analysis, five additional head-to-head RCTs (11–14) have been added, with more than 2,000 evaluable patients, but no updated reviews have been published. Moreover, in clinical practice, various dosing frequencies are used for the two taxanes, including weekly, biweekly, and triweekly regimens. The impact of dosing intervals on neurotoxicity has been formally recognized (9, 15), but whether different dosing intervals affect taxane-associated acute pain syndrome (TAPS) has not been reported in the literature. Therefore, based on the PRISMA 2020 guidelines, this study conducts a systematic review and meta-analysis comparing only nab-PTX with PTX for the first time, with a pre-specified stratification by dosing frequency. The aim is to precisely quantify the relative risk of TAPS induced by the two formulations, providing evidence-based support for clinicians to choose a less painful individualized chemotherapy regimen and ultimately improving the treatment experience of cancer patients.

## 2 Method

### 2.1 Registration

This study was prospectively registered in the prospero systematic review database, number crd42023484814.

### 2.2 Search strategy

As of November 30, 2023, we systematically searched the following databases: PubMed, Embase, and Cochrane Library. The search strategy included keywords and MeSH terms related to paclitaxel, nab-paclitaxel, etc. Finally, check to ensure no additional studies are missed. The detailed search terms and search strategy are presented in the attachment.

We conducted our research in accordance with the PRISMA guidelines. To guarantee a comprehensive search, we methodically scoured multiple databases for relevant studies, including PubMed, Embase, and Cochrane Library. Our search spanned up to November 30, 2023, ensuring the inclusion of the most up - to - date information. To enhance the search process, we combined MeSH terms or emtree terms with free - text terms. This allowed us to capture a wide array of articles that met our research criteria. The detailed search strategy, including the specific terms utilized, is presented in the attachment. Below are the relevant search terms for participants and interventions:The relevant search terms for the participants and intervention factors are as follows:

1. Patients: The term is “Neoplasms,” and the free terms are (Neoplasia) OR (Neoplasias) OR (Neoplasm) OR (Tumors) OR (Tumor) OR (Cancer) OR (Cancers) OR (Malignancy) OR (Malignancies) OR (Malignant Neoplasms) OR (Malignant Neoplasm) OR (Neoplasm, Malignant) OR (Neoplasms, Malignant) OR (Benign Neoplasms) OR (Neoplasms, Benign) OR (Benign Neoplasm) OR (Neoplasm, Benign).
2. Intervention: The drug of interest is albumin-bound paclitaxel, and the free terms are (Albumin Bound Paclitaxel) OR (Paclitaxel, Albumin-Bound) OR (Protein-Bound Paclitaxel) OR (Paclitaxel, Protein-Bound) OR (Protein Bound Paclitaxel) OR (Abraxane)) OR (ABI007) OR (ABI-007) OR (ABI 007).

### 2.3 Inclusion and exclusion criteria

The inclusion criteria are as follows:(1) Prospective phase II and III clinical trials conducted in cancer patients; (2) To calculate OR and RR, the included studies must compare nab-paclitaxel with conventional paclitaxel, or compare nab-paclitaxel with the same chemotherapy agent with the same anti-paclitaxel and the same chemotherapy agent, with the same frequency of medication; (3) The search is limited to articles and abstracts published in English; (4) Available events or incidence of muscle pain and joint pain; (5) If multiple publications of the same trial are retrieved, only the latest publication is included. This study does not require approval from the ethics committee because meta-analysis, as a secondary statistical study, has no direct relationship with the subjects.

Exclusion criteria are as follows:(1) Review articles, conference papers, meta-analyses, case reports, animal experiments, and studies that do not meet the relevant literature;(2) Literature without main outcome indicators.

### 2.4 Literature screening and data extraction

Two researchers (HBL and LW) independently read the relevant literature and extract data. If there is any disagreement, consult a third investigator (ZYW). After discovering missing data in the literature, try to contact the original author for supplementation. In the process of literature screening, the first step is to screen the title and abstract, and exclude obviously irrelevant literature; the second step is to screen the full text to determine whether it can be included in this study. Each eligible study includes the following information: the first author, publication year, treatment stage, trial design, type of cancer, median age, medication plan, medication cycle, and main outcome indicators (arthralgias, myalgias).

#### 2.4.1 Literature quality assessment

The Cochrane risk assessment tool is used to assess the risk of bias in randomized controlled studies to determine whether it affects the results. The evaluation stage is independently assessed by two researchers (HBL and LW), and finally compared and made into charts. If there is a dispute, the third researcher (ZYW) is requested to assess and discuss the decision.

#### 2.4.2 Evaluation indicators

Number of patients with arthralgias, number of patients with myalgias.

### 2.5 Statistical analysis

Meta-analysis was performed using RevMan 5.3 software. When i^2^>50%, the random effects model is used for heterogeneity test, and the odds ratio (OR) and 95% confidence interval are used for binary variables.

### 2.6 Handling of multi-arm trials

Two studies (Brufsky 2021, Zhang 2021) contained three arms (PTX, nab-PTX, and combination). Following Cochrane guidance, we selected the two relevant single-agent arms (nab-PTX and PTX) for direct comparison, following Cochrane guidance for multi-arm trials. This approach preserves the original patient counts and events without mathematical combination of different treatment arms.

### 2.7 Choice of effect measure: OR vs RR

We primarily reported odds ratios (ORs) in our main analyses. To ensure clinical interpretability and address potential overestimation of effect sizes when event rates are high, we supplementarily calculated risk ratios (RRs), which showed directionally consistent results (**Supplementary Figures S1–S3**). For clinical interpretability, we supplementarily calculated risk ratios (RRs) and present them in the supplementary figures. Both measures showed directionally consistent results, confirming the robustness of our findings.

### 2.8 Study selection process

**Figure 1:**
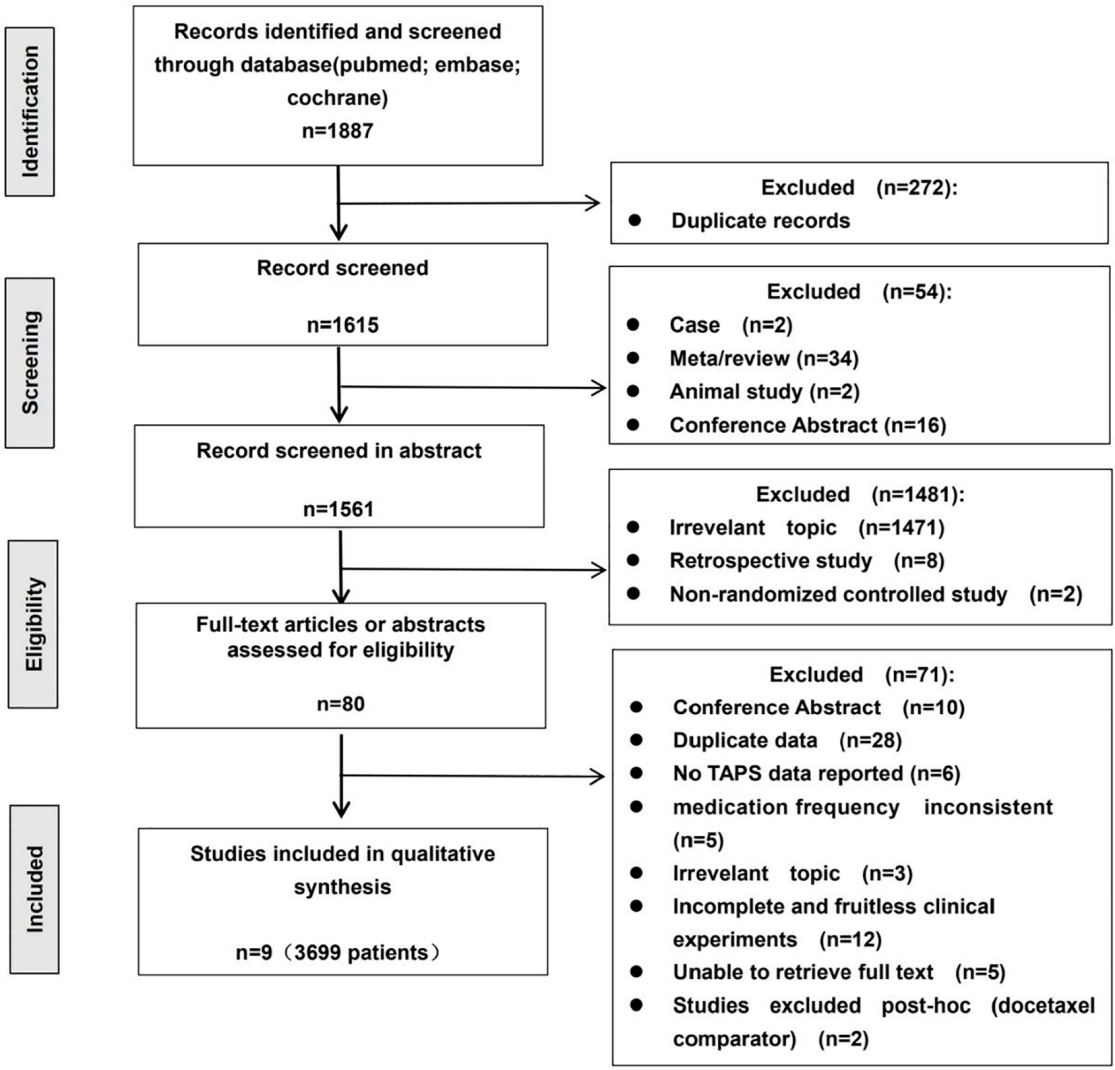
PRISMA flow diagram of study selection process. Records were identified from PubMed (n = 104), Embase (n = 191) and Cochrane Central (n = 1592). After removal of duplicates, 1615 records were screened and 80 full-text articles were assessed for eligibility. Two trials with docetaxel comparator arms were excluded *post-hoc* to restrict the analysis to paclitaxel formulations. Finally, 9 randomized controlled trials comprising 3699 patients were included in the quantitative synthesis.

We preliminarily screened out 1887 related literatures, with 104 in PubMed, 191 in Embase, and 1592 in Cochrane. Using EndNote X9 software to delete 272 duplicate publications, 1615 remaining; after the first screening through the title and abstract, 1561 publications were excluded, and 80 publications were left; according to the inclusion and exclusion criteria, after reading the full text, a total of 9 publications were included in this study. Notably, studies such as Mobus 2021 and Gradishar 2009 were excluded as they compared nab-paclitaxel to docetaxel rather than to paclitaxel (PTX). The screening process is shown in **Figure 1**.

## 3 Results

### 3.1 Inclusion of literature

**Figure 2:**
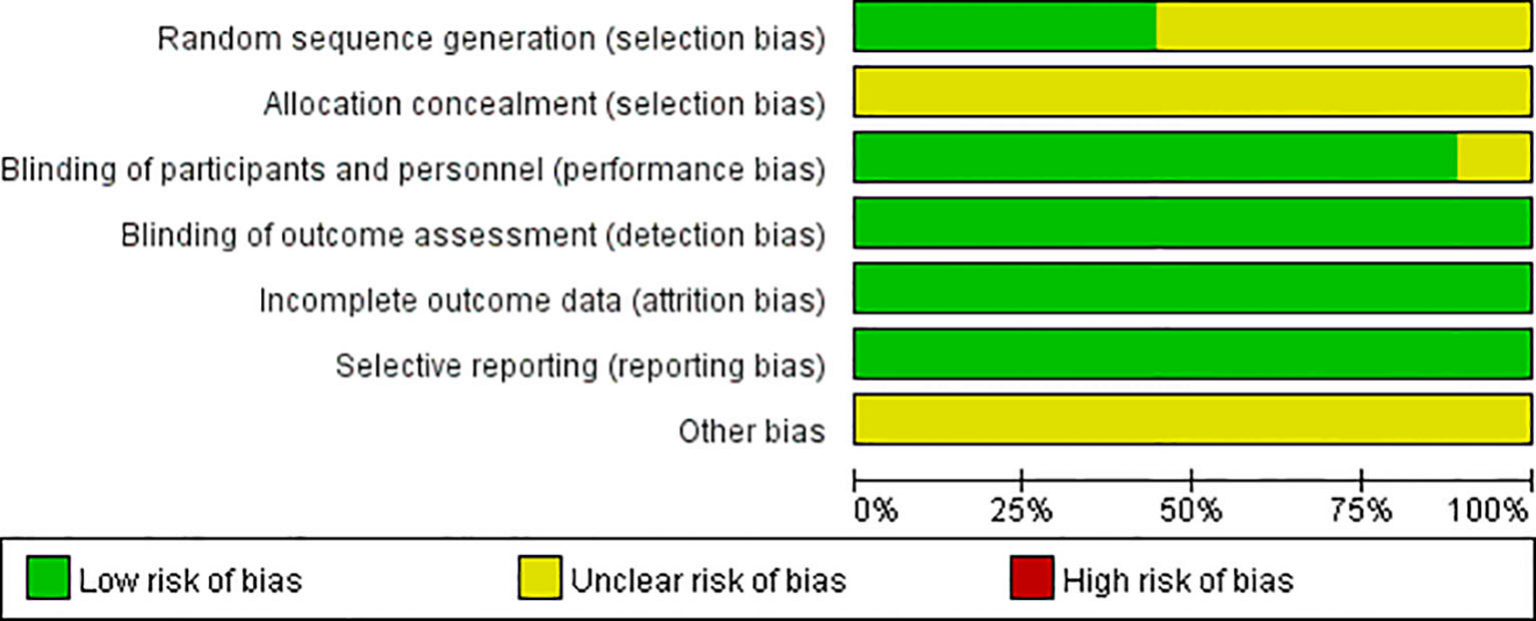
Risk of bias of included trials graph.

A total of nine randomized controlled trials (RCTs) were included, involving 3,699 patients with solid tumors. The nab-PTX group included 1,817 patients, while the PTX group included 1,825 patients. The studies covered various solid tumors, including breast cancer, gastric cancer, urothelial cancer, and non-small cell lung cancer. Specifically, six studies focused on breast cancer, with one study each on gastric cancer, urothelial cancer, and non-small cell lung cancer. Five studies had an open-label design, and all studies used NCI-CTCAE v3.0–v4.0 for pain grading assessment. All studies were published with complete data. Detailed study characteristics are shown in **Table 1**. **Figure 2** shows the risk of bias graph. The results of the risk of bias assessment are summarized in **Supplementary Figure S4**.

**Table 1:** Main characteristics of the included studies.

Author	Year	Treatment setting	Types of cancer	Type	Taxane type and dose(mg/m2)	Taxane frequency	Median age(y)	Arthralgias events(n)	Myalgias events(n)
Brufsky,A.	2021	Metastatic	Breast	RCT	paclitaxel 80	d1,8,15 q4w	52(26-79)	2/32	2/32
					nab-paclitaxel 100	d1,8,15 q4w	51(20-75)	4/31	4/31
Zhang,Y.Y.	2021	Locally advanced	NSCLC	RCT	Paclitaxel 50	d1 qw	65.5(41-87)	2/38	NR
					nab-paclitaxel 40	d1 qw	66.5(44-86)	2/37	NR
Pippen, J.	2011	adjuvant	Breast	RCT	Paclitaxel 175	d1 q2w	51.2	49/99	41/99
					nab-paclitaxel 260	d1 q2w	51.2	55/98	45/98
Sridhar SS	2020	Metastatic	Urothelial	RCT	Paclitaxel 175	d1 q3w	68 (35-84)	27/100	22/100
					nab-paclitaxel 260	d1 q3w	67 (24-88)	25/99	21/99
Guan, Z. Z.	2009	Metastatic	Breast	RCT	Paclitaxel 175	d1 q3w	48.5(27–67)	27/106	41/106
					nab-paclitaxel 260	d1 q3w	50.0(24–70)	23/104	40/104
Shitara K	2017	Advanced	gastric	RCT	Paclitaxel 80	d1,8,15 q4w	65 (59–71)	25/243	31/243
					nab-paclitaxel 100	d1,8,15 q4w	67 (60–72)	30/241	39/241
Rugo HS	2015	Metastatic	Breast		Paclitaxel 90	d1,8,15 q4w	NR	43/272	100/272
					nab-paclitaxel 150	d1,8,15 q4w	NR	43/264	111/264
Gianni L	2018	neoadjuvant	Breast		paclitaxel 90	d1,8,15 q4w	NR	130/335	NR
					nab-paclitaxel 125	d1,8,15 q4w	NR	134/337	NR
Untch M	2016	neoadjuvant	Breast		Paclitaxel 80	d1,8,15 q3w	48 (41–56)	207/601	150/601
					nab-paclitaxel 150	d1,8,15 q3w	49 (43–57)	223/605	187/605

NR, not report; RCT, randomized controlled trial; d1,8,15 q4w,administer on the 1st, 8th, and 15th days, once every four weeks; d1 qw, administer on the 1st days, once every four weeks; d1 q2w, administer on the 1st days, once every two weeks; d1 q3w, administer on the 1st days, once every two weeks; d1,8,15 q3w, administer on the 1st, 8th, and 15th days, once every three weeks.

### 3.2 Incidence of arthralgias

**Figure 3:**
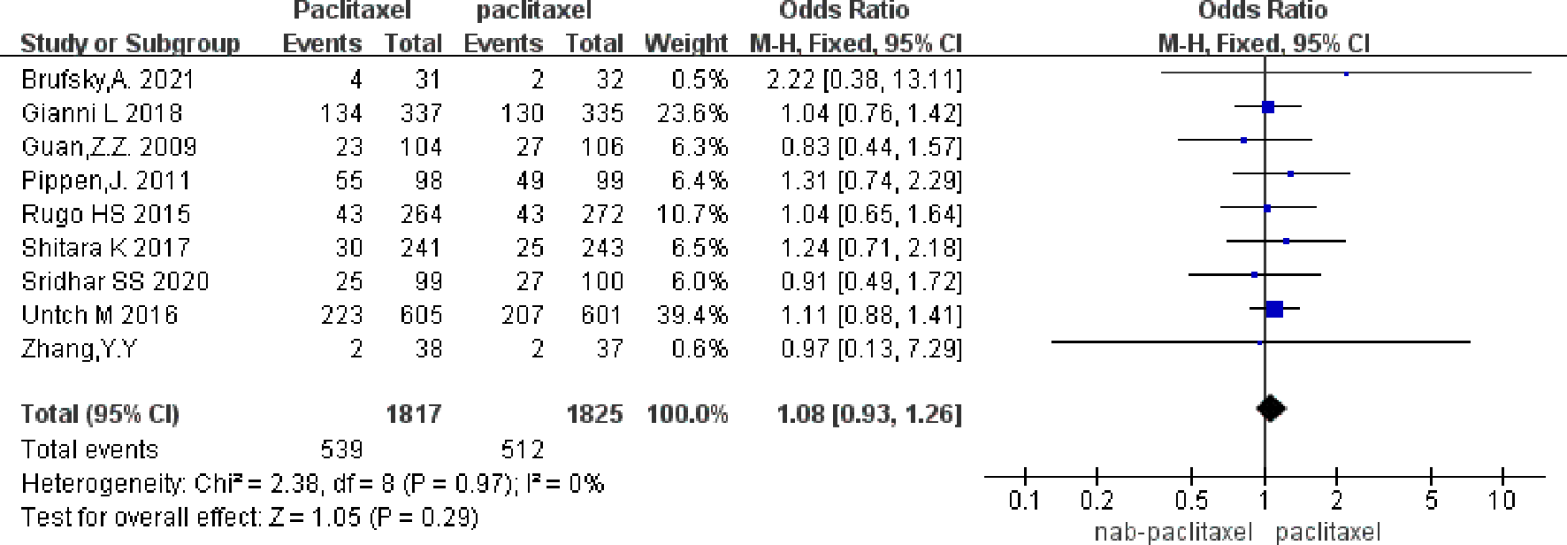
Overall arthralgias incidence results (9 studies).

Nine studies were included, involving 3,699 patients (nab-PTX group: 1,817; PTX group: 1,825). Results showed no significant difference in arthralgia incidence between nab-PTX and PTX (p = 0.29, OR = 1.07, 95% CI 0.91–1.25, I²= 0%). The forest plot is shown in **Figure 3**. The funnel plot (**Supplementary Figure S5**) showed symmetry, suggesting a low risk of publication bias.

In subgroup analysis by dosing frequency, no significant differences in arthralgia incidence were found between the q3w and q4w schedules (test for subgroup differences: p = 0.34). The point estimates varied: the q3w schedule suggested a non-significant decreased risk with nab-PTX (OR = 0.87), while the q4w schedule showed a non-significant increased risk (OR = 1.10). The 95% CIs for both subgroups crossed 1, and heterogeneity was very low (I² = 0%), indicating that the risk of arthralgia was not significantly modulated by dosing frequency.The forest plot is shown in **Supplementary Figure S6**. The funnel plot (**Supplementary Figure S7**) showed symmetry, suggesting a low risk of publication bias.

We have now performed a meta-analysis of the arthralgia subgroup using RRs. The results (RR = 1.06, 95% CI: 0.91–1.24) are consistent with the OR analysis and confirm no significant difference in arthralgia risk. This new forest plot has been added as **Supplementary Figure S1**.

### 3.3 Incidence of myalgias

**Figure 4:**
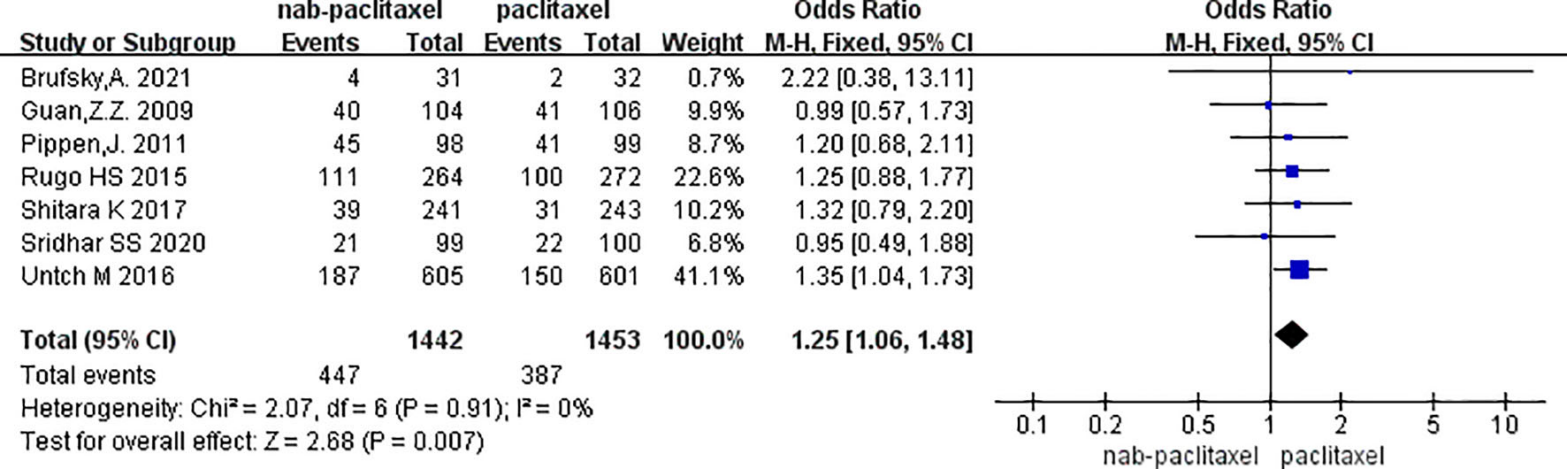
Overall myalgias incidence results (7 studies).

Seven studies were included, involving 2,895 patients (nab-PTX group: 1,442; PTX group: 1,453). Results showed a significantly higher incidence of myalgia in the nab-PTX group compared to PTX (p = 0.007, OR = 1.25, 95% CI 1.06–1.48, I²= 0%). The forest plot is shown in **Figure 4**, and The corresponding funnel plot showed no obvious asymmetry, suggesting a low risk of publication bias (**Supplementary Figure S8**).

In subgroup analysis by dosing frequency, the q4w (days 1, 8, 15) subgroup showed a significantly higher incidence of myalgia in the nab-PTX group compared to PTX (p = 0.005, OR = 1.26, 95% CI 1.06–1.49, I²= 0%). No significant difference was found in the q3w subgroup (p > 0.05, I²= 0%). The interaction test (p = 0.010) indicates a significant interaction between dosing frequency and myalgia risk.The forest plot is shown in **Supplementary Figure S9**. The funnel plot (**Supplementary Figure S10**) showed symmetry, suggesting a low risk of publication bias.

### 3.3 Subgroup analysis summary

Treatment regimen subgroup: The risk of myalgia was slightly higher in combination chemotherapy regimens compared to monotherapy, but the test for subgroup differences indicated that this difference was not statistically significant (p = 0.44).

## 4 Discussion

Taxane-associated acute pain syndrome (TAPS) is increasingly recognized for its negative impact on quality of life and treatment adherence. This systematic review and meta-analysis of 9 RCTs (11–14, 16–19) and 3,699 patients provides the first direct comparison of nab-PTX versus PTX in TAPS incidence (20). After excluding confounding taxanes, we found that nab-PTX significantly increased myalgia risk (OR 1.25, 95 % CI 1.06–1.48), while arthralgia risk was comparable. This is the first meta-analysis to systematically distinguish symptom-specific differences in myalgia and arthralgia risks between the two formulations.

We found that the increased myalgia risk was not universal but context-dependent, most pronounced in breast cancer patients, combination therapies, and weekly dosing schedules. This pattern suggests that nab-PTX myotoxicity may be related to specific tumor microenvironments, synergistic toxic effects with other chemotherapeutics, and cumulative effects of frequent dosing. In contrast, arthralgia risk showed no significant difference across all clinical contexts, constituting a robust negative endpoint—clinicians need not consider arthralgia when choosing between formulations.

Our findings differ significantly from the earlier meta-analysis by Fernandes et al., primarily because they pooled docetaxel with PTX and did not analyze myalgia and arthralgia as separate outcomes. Our granular analysis provides decisive evidence to resolve previous controversies. The dissociation between myalgia and arthralgia suggests distinct biological mechanisms: 1)Myalgia: nab-PTX utilizes gp60 receptor-mediated transcytosis, potentially leading to higher distribution and retention in muscle-rich tissues (20). Its nanoparticle properties may facilitate capillary wall passage, directly acting on muscle cells to trigger intense local inflammation or mitochondrial dysfunction (21, 22). Combination therapy and weekly dosing may exacerbate this through additive toxicity or reduced tissue repair time. 2)Arthralgia: more likely mediated by systemic inflammatory factors, with both formulations exerting comparable stimulation (13, 14). Alternatively, as a deeper-seated pain, it may overlap more with neuropathic pain mechanisms, where both formulations have similar efficacy (13, 14).

Notably, the increased myalgia risk was primarily associated with the every-4-week (q4w, days 1, 8, 15) dosing schedule, with a 21% higher incidence compared with PTX (interaction p = 0.011). Clinically, the q4w nab-PTX regimen is widely used for its high dose intensity and short infusion time. However, our data indicate that this convenience comes at the cost of increased acute pain. Up to one-quarter of patients receiving q4w nab-PTX experienced moderate-to-severe myalgia (11, 14), consistent with individual trial reports (11). This risk should be weighed against expected efficacy benefits, especially in curative or adjuvant settings where treatment discontinuation could compromise outcomes. Practical strategies include prioritizing q3w or weekly schedules in pain-vulnerable patients or using routine dexamethasone prophylaxis (8 mg daily × 3 days), which has shown dose-dependent benefits in recent phase II trials (23).

Mechanistically, higher peak concentrations of unbound paclitaxel after q4w nab-PTX may trigger Toll-like receptor 4 signaling and acute neuroinflammation, although direct neural penetration data are lacking (20). The absence of Cremophor in nab-PTX may also facilitate endothelial transcytosis, increasing neural exposure (15). These hypotheses require translational validation but align with observed schedule-dependent toxicity.

Strengths include exclusive comparison of nab-PTX vs PTX, low heterogeneity, pre-specified subgroup analyses, and concurrent reporting of OR and RR. The robustness of our primary findings, including the key subgroup finding of increased risk with the q4w schedule, is supported by consistent results in sensitivity analyses using risk ratios (see **Supplementary Figures S8–S10**). Limitations include predominance of open-label trials, potential detection bias for subjective pain endpoints, heterogeneous CTCAE versions (v3–v4), and lack of quality-of-life data.

In conclusion, compared with PTX, nab-PTX shows a modest but consistent increase in myalgia risk, primarily within the q4w dosing schedule. Oncologists should integrate this evidence into treatment planning, considering alternative dosing schedules or proactive analgesia when nab-PTX is preferred. Prospective, blinded trials using patient-reported outcome measures are needed to confirm the optimal risk-benefit balance of different nab-PTX regimens.

## 5 Conclusion

In nine randomized trials, nab-PTX was associated with a significantly increased incidence of taxane-associated acute myalgia compared with PTX. This increased risk was predominantly driven by the every-4-week (days 1, 8, 15) schedule, while overall arthralgia rates were similar. Dosing frequency rather than cumulative dose appears to drive this difference. Clinicians should consider dosing schedule selection and routine prophylactic analgesia to mitigate TAPS when using nab-PTX.

## Data Availability

The original contributions presented in the study are included in the article/**Supplementary Material**. Further inquiries can be directed to the corresponding author.
