# Supplementary material for: Nab-paclitaxel versus paclitaxel for taxane acute pain syndrome in solid tumors: a systematic review and meta-analysis
Source: Front Oncol. 2026 Jan 14;15:1650191. doi: 10.3389/fonc.2025.1650191 (PMC12846926; doi:10.3389/fonc.2025.1650191)
Supplement: Supplementary file 1 [file DataSheet1.docx]

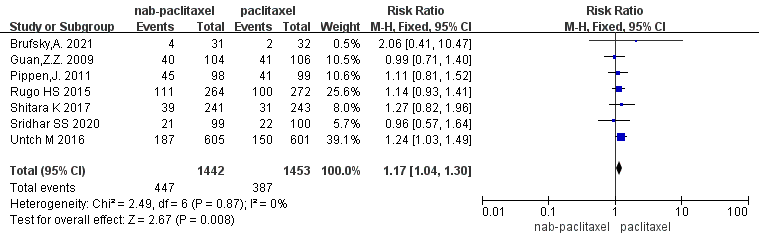

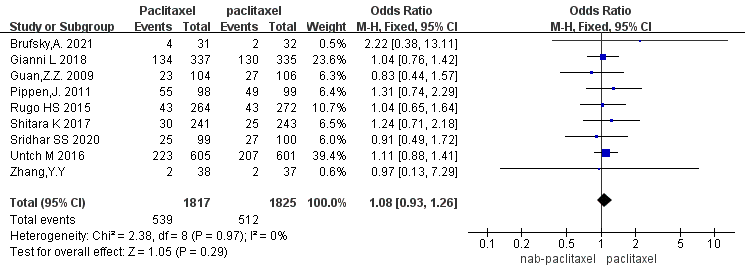

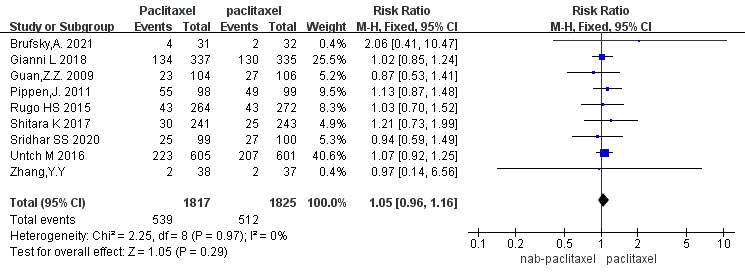


Supplementary Figure S1. **Risk ratio (RR) forest plot for the overall incidence of arthralgia** (9 studies).


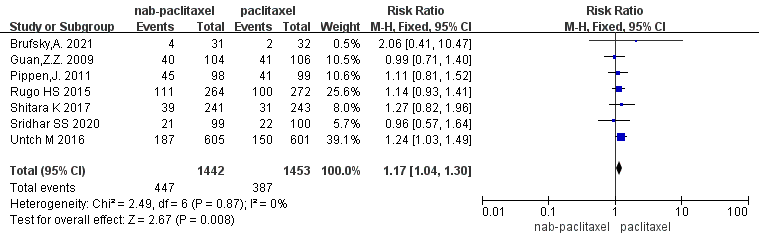


Supplementary Figure S2. **Risk ratio (RR) forest plot for the overall incidence of myalgia** (7 studies).


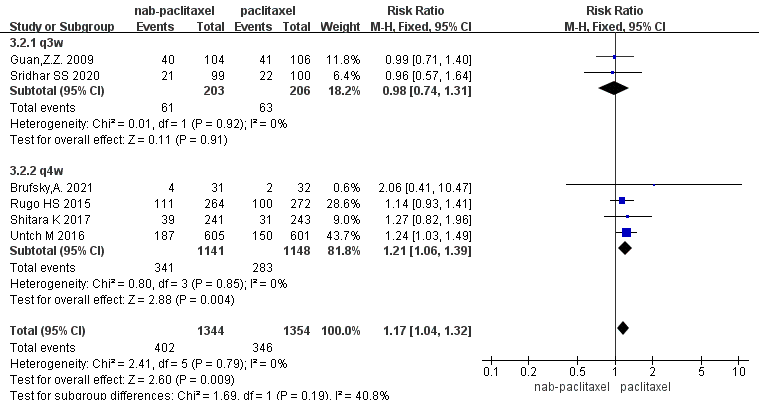


Supplementary Figure S3. **Risk ratio (RR)** of nab-paclitaxel to paclitaxel causing myalgia at different medication frequencies.


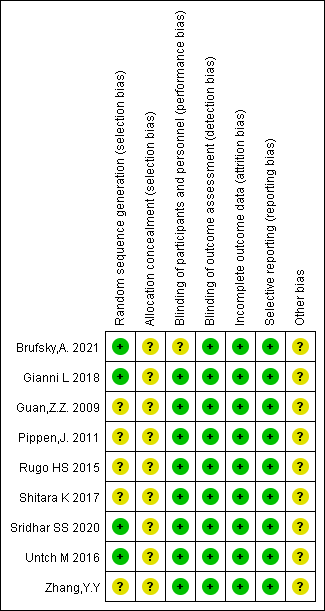


Supplementary Figure S4. **Risk of bias summary for the included randomized controlled trials.**
The assessment was performed using the Cochrane Risk of Bias tool (RoB 2.0). Review authors' judgements about each risk of bias item for each included study are presented. Green circle (+): low risk of bias; Yellow circle (?): unclear risk of bias; Red circle (-): high risk of bias.


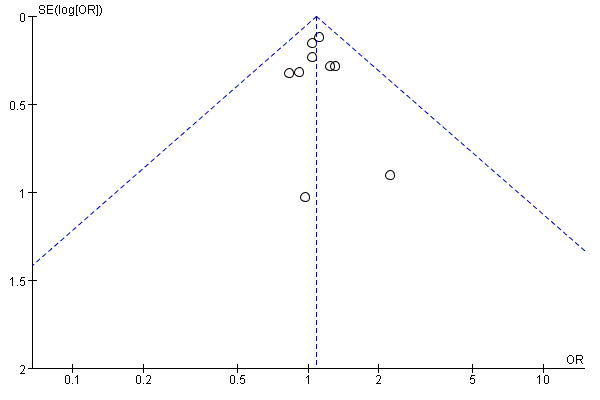


Supplementary Figure S5. Funnel plot for the overall analysis of arthralgia incidence. The symmetrical distribution of studies suggests a low risk of publication bias.


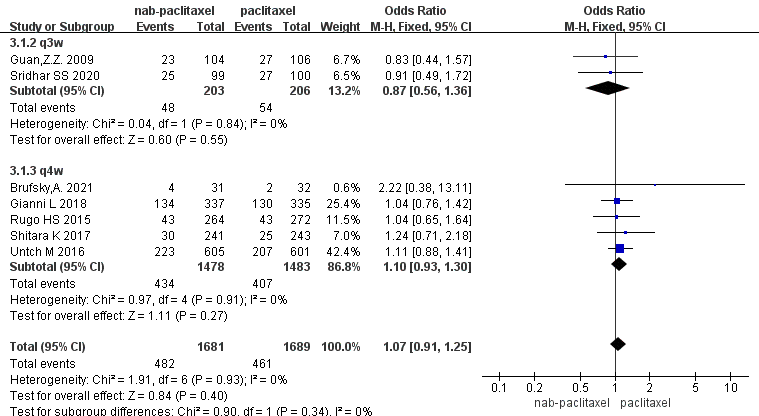


Supplementary Figure S6. Odds ratio of nab-paclitaxel to paclitaxel causing arthralgias at different medication frequencies.


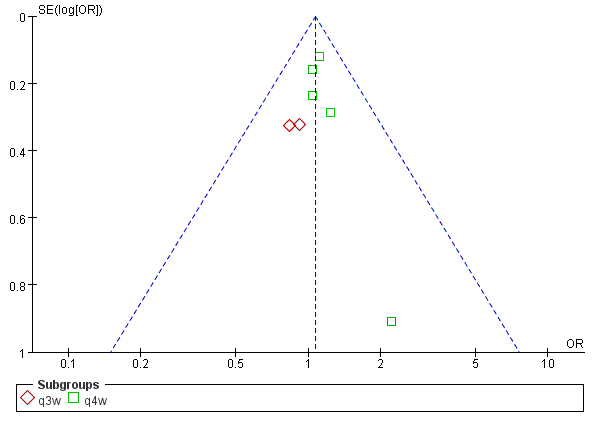


Supplementary Figure S7. The funnel plot showed that there was low publication bias.


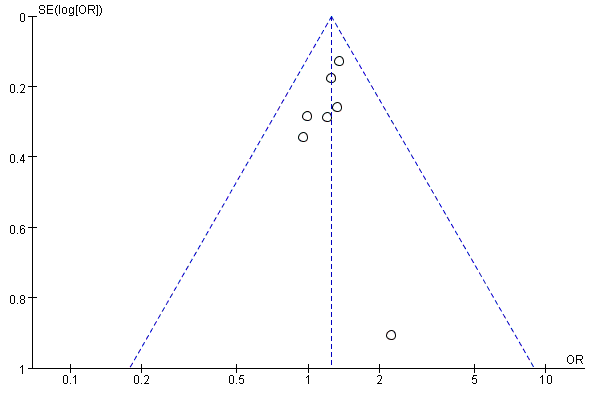


Supplementary Figure S8. The funnel plot showed that there was low publication bias.


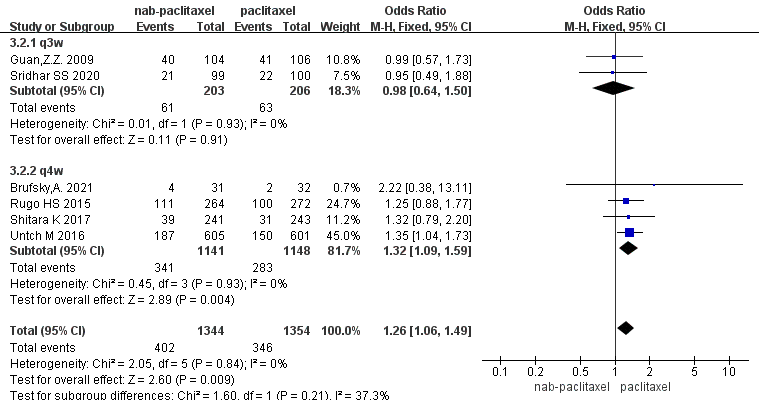


Supplementary Figure S9. Odds ratio of nab-paclitaxel to paclitaxel causing myalgia at different medication frequencies.


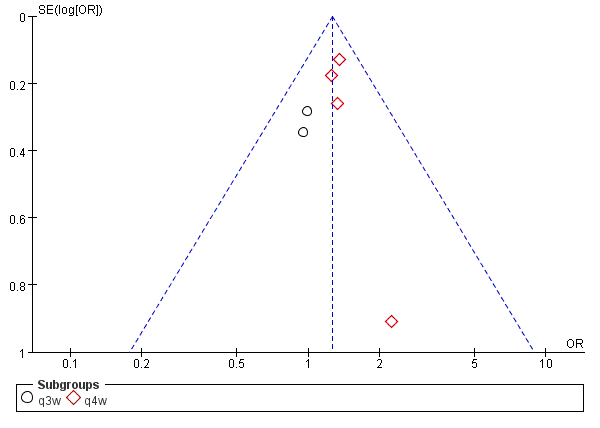


Supplementary Figure S10. The funnel plot showed that there was low publication bias.
